# The effects of the SARS-CoV-2 pandemic on children and youth with special health care needs

**DOI:** 10.3389/fped.2022.1007770

**Published:** 2023-01-06

**Authors:** Mel Michaud, Irene Cihon Dietz

**Affiliations:** ^1^ School of Medicine, Case Western Reserve University, Cleveland, OH, United States; ^2^ Department of Pediatrics, The MetroHealth System, Cleveland, OH, United States; ^3^ Case Medical Center, School of Medicine, Case Western Reserve University, Cleveland, OH, United States

**Keywords:** COVID-19, children with disabilities, children with special health care needs, CYSHCN, pandemic

## Abstract

This article seeks to review the current knowledge of the SARS-CoV-2 virus and the health effects for children and youth with special health care needs (CYSHCN). COVID-19, an infectious disease caused by the severe acute respiratory syndrome coronavirus 2 (SARS-CoV-2), became a major pandemic in 2020. Recognition of the disease could be difficult, as symptoms in children are at times different than adults and can mimic other common childhood viral infections. Children with underlying medical conditions did make up a higher proportion of those hospitalized, but also were affected in other ways including loss of nursing support, missed education and rehabilitative services, and increased stress for themselves and their families, affecting mental health in this vulnerable population.

This review seeks to address what is currently known about the overall effects on CYSHCN and their families, and identify gaps in research, including the implementation of health care systems, and possible suggestions for change in the educational and community supports for this group of individuals. Ongoing analysis of large national and international data sets, as well as smaller reports based on specific congenital anomaly, genetics disease, and acquired childhood illness, and then attention to local resources and family resilience is still necessary.

## Introduction

Children and Youth with Special Health Care Needs (CYSHCN) is defined by the federal Maternal and Child Health Bureau as “those who have or are at increased risk for a chronic physical, developmental, behavioral, or emotional condition and who also require health and related services of a type or amount beyond that required by children generally” (1). In the 2009/2010 National Survey of CYSHCN, approximately one in five families has at least one child with special health needs which translates into approximately 14.6 million children (1). As of July 22, 2022, more than 90.2 million COVID-19 cases and more than 1.02 million deaths have been reported, according to Johns Hopkins University (2). Among children, more than 13.9 million cases have been reported as of July 14, 2022, according to American Academy of Pediatrics data (3). Using these estimates, nearly 2.9 million CYSHCN have been ill with COVID19. But even for those that did not have the infection, major life changes have occurred related to social isolation, school closing, unavailability of in home and school nursing and therapy supports and shift to telehealth or video only visits. While the rapid shift to telehealth was amazingly effective for families with proper internet and computer or smart phone access, again underserved and vulnerable communities lack these resources. Research specific to CYSHCN has been slower than anticipated as the pandemic has evolved. Information presented is the result of both a PubMed literature search using key-words SARS-CoV-2 and CYSHCN, and well as COVID-19 and CYSHCN performed on March 31, 2022; and again July 22, 2022, reveals with the results of a recently published literature search for the effects of Sars-Cov-2 in nearly 3,000 individual adults with genetic and congenital conditions by Hromic-Jahjefendic et al.

## Recognizing COVID-19 symptoms in CYSHCN

Early in the pandemic, the disease was clearly recognized to be more likely to cause death in the elderly, or those with conditions such as obesity, diabetes mellitus, or chronic pulmonary disease (COPD), as well as cause difficulty in access to ongoing medical care for those with cancer, or “non-emergency” surgical needs. Chen and colleagues report on the first 799 people with the disease who were admitted to the isolation ward of a hospital in Wuhan, China, assigned for patients with severe or critical covid-19. The authors compared the characteristics of 113 (14.4%) patients who had died thus far with those of 161 patients who recovered, finding that those who died were on average 17 years older (with no deaths among those aged under 40% and 16.8% of deaths among those aged 40–60), more likely to be male, and more likely to have a comorbidity such as hypertension, diabetes, cardiovascular disease, or chronic lung disease (4). For many this supported the hypothesis that the disease itself was mild in children. or incorrectly assumed “children don't get COVID19.”

Data began to mount that children were clearly not immune to the disease and could in fact have severe outcomes. Pediatricians were faced with difficulty identifying symptoms of COVID- 19 and testing for the virus when a child was presented with many of the same complaints as other common childhood illnesses like influenza, respiratory syncytial virus, or viral rashes. Other symptoms could include emesis or diarrhea, with minimal respiratory issues. Together with sometimes vague and unrecognized symptoms, limited readily available testing resources were lacking for almost the first year of the pandemic.

While the Center for Disease Control (CDC) turned public attention to identifying at risk adults, and “flattening the curve” of rapid viral spread with use of universal personal protective equipment in hospitals and health care setting, isolation, and masking in the general population, information about COVID-19 infections in children was initially less scrutinized. Severe cases and deaths were mostly reported in the elderly, and those with chronic medical illness. Information about COVID-19 infections in children did emerge a few months later, showing that like adults, children with underlying medical conditions, or CYSHCN, were more likely to be infected. The limited supply of testing materials led to only those sickest persons presenting in emergency rooms and being considered for admission having COVID-19 or viral testing.

Businesses, schools, medical providers including Pediatricians were directed to look for respiratory symptoms and fever as screening for a clearance to participate in one's usual daily activities. But as the pandemic progressed, we discovered that many of the same symptoms could be attributed to other common childhood illnesses like Group B streptococcus pharyngitis, Influenza Type A or B, and Respiratory Syncytial Virus (RSV) bronchiolitis. For children with medical complexity with underlying intellectual disability, epilepsy, chronic respiratory concerns, feeding tube dependence and chronic constipation, it was difficult to determine if a child presenting with increased emesis and seizure frequency was in fact COVID19 or severe constipation, gastroesophageal reflux (GERD), tube malfunction or another etiology. Providers familiar with care of children with medical complexity (CMC), a subset of CYSHCN, and with the individual patient may have been in a better position to determine if the signs were more consistent with underlying medical condition, but again early on these offices lacked testing supplies. Only the sickest individuals were to be referred to the emergency rooms, and many visits were changed to virtual or telehealth only.

Parcha et al. completed an analysis of 12,306 children from the United States infected with COVID-19 from April-October 2020 in TriNetX database. Only 25.1% of children had at least one of the typical symptoms (fever, cough, or shortness of breath), and 9.9% of children had at least two typical symptoms (5). Three-fourths (74.9%) of the children did not have any of the typical COVID-19 symptoms (5). The symptoms recorded for the children included respiratory (16%), gastrointestinal (13.9%), rash (8.1%) neurological (4.8%), but also nonspecific findings such as fever, malaise, sore throat, runny nose, sneezing, fatigue (18%) (5). Only 5% required hospitalization, of whom 17.6% needed mechanical ventilation (5).

## Underlying medical conditions associated with higher risk for COVID-19 infection in children and CYSHCN

**Figure 1:**
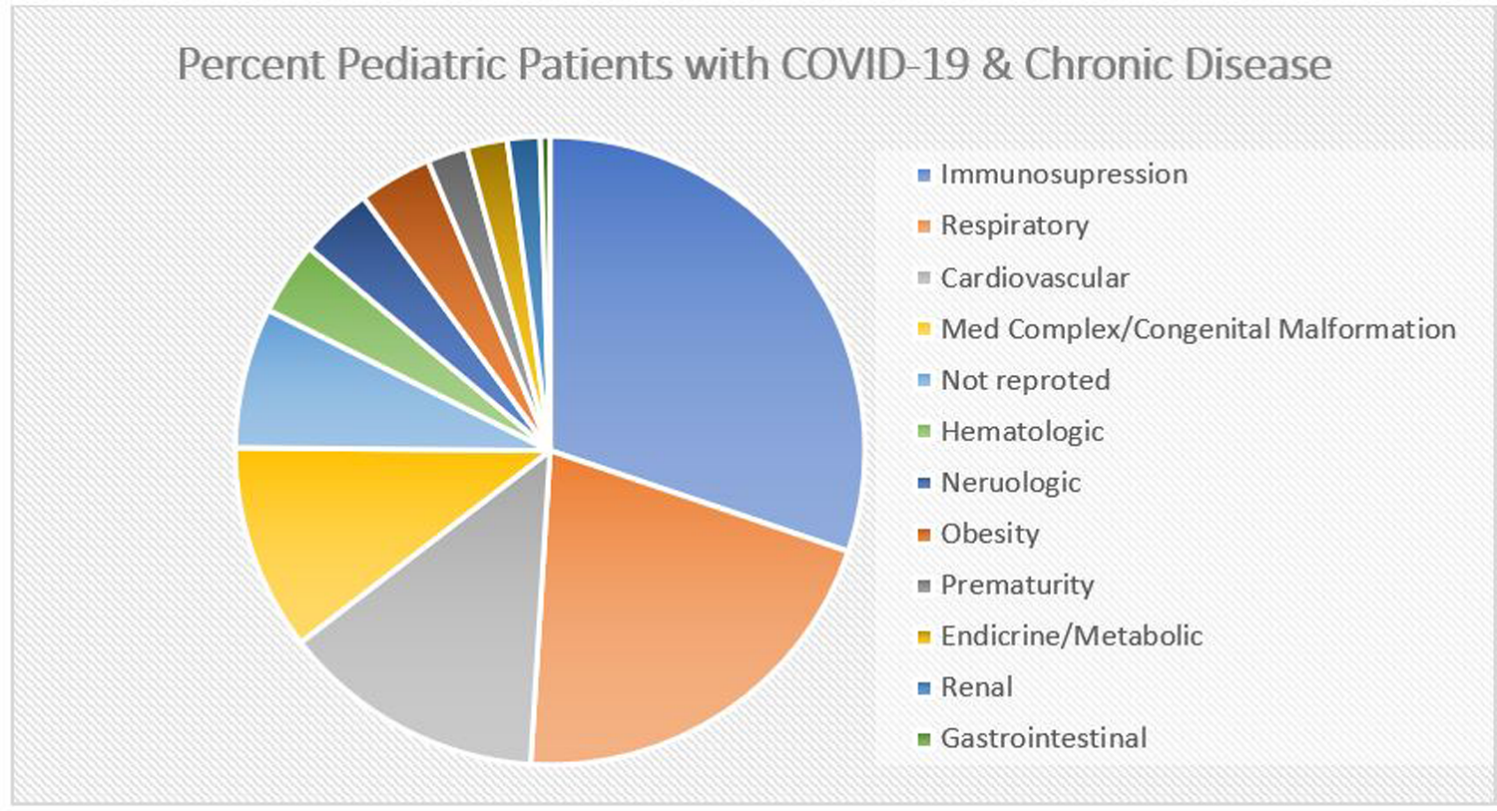
Adapted from Hoang et al. (6), demonstrates the percentage of each chronic condition reported in 655 children with COVID-19 from 20 studies.

Hoang et al. performed a meta-analysis/case summary of children with COVID-19, looking at 131 studies from 26 countries within the first 6 months of the pandemic. This included 7,780 children from January to May 2020, with 2,572 children from the United States and 64 (1%) of children from China. Just 20 studies (*n* = 655 individuals) reported an underlying medical condition; COVID-19 positive children who were immunosuppressed or had a history of a respiratory or cardiac condition comprised the majority (65%) (6) (Figure 1).

**Figure 2:**
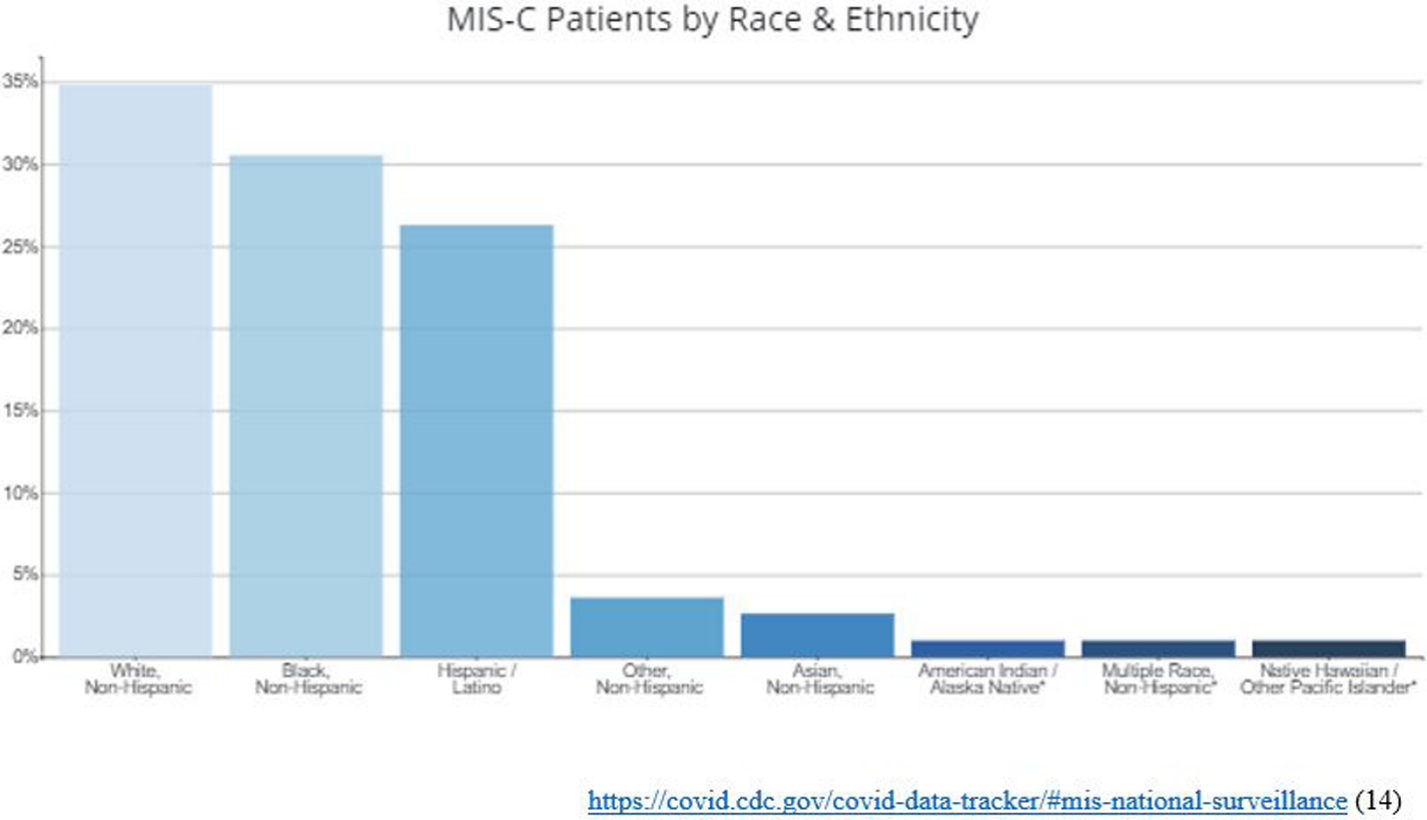
The percentage of patients with MIS-C by Race and Ethnicity reported through the local Health Departments to the CDC. The median age of patients with MIS-C was 9 years. Half of children with MIS-C were between the ages of 5 and 13 years. 53% of the reported patients with race/ethnicity information available (N=8,607) occurred in children who are Hispanic/Latino (1,919 patients) or Black, Non-Hispanic (2,618 patients).

Bailey and colleagues looked at a collection of data from PedsNet, a network of 7 US pediatric health systems, comprising 6.5 million patients primarily from 11 states. This resulted in 135,794 patients younger than 25 years who were tested for SARS-CoV-2 from Jan 1-Sept 8, 2020. Testing for SARS-CoC-2 was considered the exposure, and the main outcomes collected were testing positive for infection, and then actual symptomatic illness. Demographics of the tested group were 59% white, 15% Black, 11% Hispanic, 3% Asian patients, and 5,374 (4%) had documented infection with the virus. Black, Hispanic, and Asian race/ethnicity had lower rates of testing. But they were significantly more likely to have positive test results (Figure 2). This data set also demonstrated that like adults, people under age 25 with underlying illness or chronic conditions, therefore likely to be considered CYSHCN, had a higher risk of positive test results at the following rates (from highest to lowest): gastrointestinal disorders, malignant disorders, endocrinologic disorders, metabolic disorders, hematologic disorders, mental health disorders, genetic disorders, musculoskeletal disorders, and cardiac disorders (7).

Most of the data we have to date is retrospective, and from large databases that may not represent all areas in terms of diversity, race and ethnicity, or socioeconomic status, and these rely on billing codes to try to tease out chronic medical conditions that existed in the patient before COVID-19 infection and those more likely to be a common complication associated with COVID-19. Studies from January 2020 through March 2021 likely reflect the Delta variant. Kompaniyets et al. completed a cross-sectional study including patients aged 18 years and younger with International Statistical Classification of Diseases, Tenth Revision, Clinical Modification code U07.1 (COVID-19) or B97.29 (other coronavirus) during an emergency department or inpatient encounter from March 2020 through January 2021, They utilized the Premier Healthcare Database Special COVID-19 Release (PHD-SR) (release date, March 15, 2021), a large, hospital-based, all-payer database which collected data from over 800 US hospitals. The study included 43,465 patients with COVID-19 aged 18 years or younger, median (interquartile range) age was 12 (4–16) years; 22,943 (52.8%) were female patients; 12,491 (28.7%) had underlying medical conditions (8).

To identify those children with medical complexity and those without from the database, the authors used the validated Pediatric Medical Complexity Algorithm (PMCA) to divide the group into those with complex chronic disease, non-complex chronic disease, or absence of chronic disease groups (9). Using the PCMA, Kompaniyets et al. concluded that the most common underlying conditions in order were asthma, neurodevelopmental disorders, anxiety and fear-related disorders, depressive disorders, and obesity. Children with cardiac and circulatory congenital anomalies, essential hypertension, and type 1 diabetes had higher risk of both hospitalization and severe illness when hospitalized. Prematurity was a risk factor for severe COVID-19 illness among children younger than 2 years. The strongest risk factors for hospitalization were type 1 diabetes and obesity, however, type 1 diabetes and cardiac and circulatory congenital anomalies were the strongest risk factors for more severe illness (8). Williams et al. also did a systematic review to try to identify and describe which underlying comorbidities were associated with severe SARS-CoV-2 disease and death. The systematic review identified 1,726, of which only 28 studies fulfilled the inclusion criteria. 5,686 participants with confirmed SARS-CoV-2 infection ranging from mild to severe disease. Of these patients, only 108 pediatric patients with severe/critical illness required ventilation, and of these, medical history was available for 48 patients (10). Thirty-six of the 48 patients (75%) had documented comorbidities of which 11/48 (23%) had pre-existing cardiac disease (10). Only 17 patients died, with past medical history was reported in just 12 cases (10). Of those, 8/12 (75%) had co-morbidities (10).

All the above studies relied on data collected early in the pandemic, thus mostly reflecting the Delta variant. Delta was found to be more likely to cause serious illness in the elderly. The next wave of illness involved a series of Omicron variants that emerged in Summer/Fall 2021 in the United States. In March 2022, the American Academy of Pediatrics offered new Guidelines for Caring for Children after COVID-19 infection More children under the age of 5 years were hospitalized at the peak of Omicron 15/100,000 than during the peak of Delta, just 3/100,000. Infants less than 6 months were hospitalized at a rate of 68/100,000 during Omicron compared to 11/100,000 during Delta, or about %X (11). This did not provide more information about CYSHCN specifically.

## Severe COVID-19, multisystem inflammatory syndrome in children (MIS-C) and long COVID-19 with CYSHCN

The spectrum of COVID-19 clinical manifestations is variable and fairly broad. Individuals infected with SARS-CoV-2 (those with COVID-19 disease) can present as asymptomatic or with mild symptoms such as fever, fatigue, sore throat, runny nose, and coughing. Severe COVID-19 develops in some individuals and is characterized by interstitial pneumonia, hypoxemia, and acute respiratory distress syndrome (ARDS), which may be lethal. Woodruff et al. examined the COVID-19—Associated Hospitalization Surveillance Network During March 2020 to May 2021 and identified 3,106 children hospitalized with laboratory-confirmed severe acute respiratory syndrome (ARDS) coronavirus 2 infection in 14 states. Among 2,293 children primarily admitted for COVID-19, multivariable generalized estimating equations generated adjusted risk ratios (aRRs) and 95% confidence intervals (CIs) of the associations between demographic and medical characteristics abstracted from patient electronic medical records and severe COVID-19. Approximately 30% of hospitalized children had severe COVID-19; 0.5% died during hospitalization. Among hospitalized children aged <2 years, the following risk factors were associated with severe COVID-19: chronic lung disease, neurologic disorders, cardiovascular disease, prematurity, and airway abnormality in descending order. Among hospitalized children aged 2–17 years, feeding tube dependence, diabetes mellitus and obesity were associated with severe COVID-19. Severe COVID-19 occurred most among infants, 12 per 100,000 children overall. Hispanic children, and non-Hispanic Black children also had higher rates of severe COVID-19 (12).

It was ultimately recognized that while many children may have milder symptoms with the infection, weeks after recovering some of these children developed a hyperimmune, post-inflammatory state can lead to a Kawasaki type syndrome. This hyperimmune state was designated as multisystem inflammatory syndrome in children (MIS-C) in May 2021 with the Center for Disease publishing standard criteria for this diagnosis. MIS-C presents as high fever, and then rapid life—threatening multisystem organ failure. Persistent fever, conjunctivitis, skin rash, myocardial dysfunction, hypotension or shock and temporary development of coronary artery dilatations are common clinical complications associated with MIS-C. These features overlap with symptoms of Kawasaki disease, a febrile inflammatory and systemic vasculitis of unknown etiology that leads to coronary artery aneurysms in young children (13).

Tracking of MIS-C in the United States by the CDC indicates that at least one case has been reported from each of the 50 states and additional territories, including Puerto Rico, Guam, U.S. Virgin Islands, Washington DC. Black race/ethnicity, Hispanic/Latino and Non-Hispanic Black populations are also disproportionately affected by COVID-19. Additional studies of MIS-C are needed to learn why certain racial or ethnic groups may be disproportionately affected, and to understand other risk factors for this disease (14). Data about underlying additional special health needs in has not been fully studied, but children with hyperimmune or autoimmune states appear to be at the highest risk. Treatment recommendations are for Pediatric Intensive Care Unit (PICU) for necessary respiratory and organ supportive measures including fluid resuscitation ventilators and inotropic medications. In rare cases, extracorporeal membranous oxygenation (ECMO) has been necessary for support. Anti-inflammatory measures have included the use of IVIG and steroids. The use of other anti-inflammatory medications and the use of anti-coagulation treatments have been variable. Aspirin has commonly been used due to concerns for coronary artery involvement, and antibiotics are routinely used to treat potential sepsis while awaiting bacterial cultures. Thrombotic prophylaxis is often used given the hypercoagulable state typically associated with MIS-C (14).

The most recent study was done in April 2022 by Hromić-Jahjefendić et al. which looked at the amount of hits in PubMed using specific genetic and congenital disorders plus COVID-19 as keywords and ranking these by how many hits they found. The most common relationship that they found was with congenital heart disease and COVID-19. Other conditions that made the top five with relationship to COVID-19, in order, were Cystic Fibrosis, Autism Spectrum Disorder, Autoimmune Hemolytic Anemia, and Hemophagocytic Lymphohistocytosis. While this furthered the relationship of these diseases with COVID-19 in some way, the only disease where children were specifically mentioned was in the discussion about Autism Spectrum Disorder (ASD). Families with children with autism primarily reported behavioral/mental health issues related to disruption of routine, and lack of school supports (15).

## Impact on access to medical and intervention services

Access to services for all children, and specifically CYSHCN, has typically emphasized coverage, service, timeliness, and capability. Services such as multiple specialty physician visits, school and home health nurse support, durable medical equipment such as ventilators, wheelchairs and orthotics, and increased hours of parental hands-on daily care are ubiquitous for these families. In home therapies such at Early Intervention, and school based rehabilitative services rapidly changed even disappeared for these families. Even with some areas having continued traditional in person medical clinics or where schools were open, most guardians were fearful of leaving their home and seeking evaluation in the office and emergency room, where the highest cohort of sick individuals and exposure to active disease was most prevalent. Most CYSHCN receive habilitation/rehabilitation services as a part of their school curriculum. Rather than being immune to other disparities in services, often these children and their families create a special group of marginalized citizens, with the issues of poverty or low socioeconomic status, race and ethnicity and lack of insurance. The closing of schools resulted in a lack of school-based health care and therapies in addition to education. While there has been much research due to the rapid and ease of transmission of the COVID-19 virus, many rehabilitation providers (PT (Physical Therapist), OT (Occupational Therapist), and Speech for example), moved to remote or canceled sessions all together. This created a higher burden on the families to be more participatory in the child's rehab services, and that required access to internet and electronic devices (16). Evaluations of the outcome of this rapid loss of services and then loss or slowed achievement of major milestones are difficult to measure for each individual child; however, there are some studies that show overall generalities.

According to a study by Allison et al. 42% of children with disabilities lost access to therapy services and 34% received services *via* telehealth. While there is convincing evidence promoting telehealth as a reasonable alternative if in person therapy is not available, it is still difficult to continue the same types of therapy. In this study, children were found to experience a dramatic loss of services, and therefore had a decline in functioning. This was especially true if the child was medically complex. “Children receiving a greater number of services pre-COVID-19 and having access to more technological devices pre-COVID-19 were significantly more likely to receive teletherapy” (17). Overall, virtual therapy during the pandemic was challenging and caused both slowing of new skills achievement, and for some children, setbacks in their development.

## Impact on CYSHCN and their families mental health

It has been well documented that the COVID19 pandemic itself as well as the steps taken to mitigate spread has had a profound impact on the mental health of children and adolescents. School closures and stay at home mandates have caused increased isolation which increases anxiety and depression. However, CYSHCN did not appear from the pandemic unscathed by the current increase in mental and behavioral health concerns. A study done by Guller et al. surveyed 299 children and adolescent with neurodevelopmental disorders like autism spectrum disorder and intellectual disability as well as their families and asked about the child's emotional, behavioral, and sleep problems as well as their appetite changes during the pandemic. Of the parents surveyed, 44.5% stated that their child has emotional problems, 33.4% behavior problems, 65.2% had sleep problems, and 32% had appetite problems. Irritability and hyperactivity were the highest reported behaviors by these parents (18).

Another study by Montirosso et al. surveyed 1,472 families in Italy about their children with neurodevelopmental disorders, and using the parent-report Child Behavior Checklist (CBCL) which was modified to ask if their child's behavior across the original CBCL dimensions (emotional reactivity, anxiety/depression, sleep problems, somatic concerns, withdrawn behavior, attention concerns, and aggressive behavior concerns) were decreased, the same, or increased pre- to post-COVID19 across a 5 point scale (19). The surveys showed a significant increase in behavior regulation in children/adolescents compared to pre-pandemic, specifically in the anxiety/depression, attention problems, and aggressive behavior dimensions. This dysregulation is displayed in the increase in symptoms such as clinginess, inattention, and irritability (19). Another study done by Masi et al. surveyed caregivers of children with neurodevelopmental disability on their child's symptom severity and well-being. This study found that, like Montirosso's study, their children were more easily annoyed, irritable, and angry since COVID-19 and the children were having difficulty maintaining relationships. They also found that approximately 20% of caregivers reported an increase in their child's medication (20). Overall, the COVID-19 pandemic had a negative impact on CYSHCN's mental health.

The CYSHCN are not the only ones who had difficulty with coping with the COVID19 pandemic. There is also research done on the overall stress levels and mental health of the caregivers of CYSHCN. In Masi's study mentioned above, they also surveyed the caregivers about their own mental health and well-being and found that 76.1% reported that COVID-19 has had an impact. Also, 73.6% reported difficulty balancing work with childcare/family responsibilities. 43.4% of parents reported an exacerbation of an existing mental health condition (20).

Another study performed by Willner et al. compared the mental health of caregivers of children with intellectual disabilities and caregivers of children without intellectual disabilities in the UK. They found that caregivers of individuals, particularly children, with intellectual disability had a 5-fold increase in the rate of severe anxiety and a 4 to 10-fold increase in the rate of major depression, compared to parents whose children did not have intellectual disability. Factors that were found to relate to the reasons why this increase was seen include more challenging behaviors from the child, increased financial pressure, and less social support (21).

However, there are some resilience factors that have been studied that show protective effects against the mental health impacts of COVID-19. In the study cited above by Monirosso et al. factors such as a positive view of the future or hope and higher perception of self, which is described as understanding one's child and their self-limitations and working within the parents’ capabilities to help themselves and their child, lead to increased resilience factors. These factors have been shown to lower their capacity to score high in anxiety and depression during the COVID pandemic (19). In another study by Yusuf et al. it was determined that the resiliency of a family/child during the pandemic relied on three factors: type of diagnosis, parenting self-efficacy, and ease in accessing schooling. This study looked at multiple domains of functioning, including nutrition and access to school. The study found that if a parent could help their child cope with the pandemic including getting them access to schooling and finding “silver lining” activities to do with their child, they were most likely to fall into what the researchers called a “Resilient profile” and they had limited decreases across all domains (22).

## Vaccine hesitancy in CYSHCN

The COVID19 vaccine was approved for use in the US in late 2020. As of 8/31/2022, approximately 79.2% of the US population have had at least one dose of the COVID-19 vaccine while approximately 67.5% of the US population is fully vaccinated with the initial series according to the CDC (23). COVID-19 vaccine has a particular increase in hesitancy given the perception that it was developed more quickly than other vaccines. The overall populations vaccine hesitancy with the COVID-19 vaccine is high. According to a commentary by Overhage, et al. in July 2021, there were 33% of Americans who were not eager to get the vaccine. The groups of people most represented in this group included young adults, women, non-Hispanic black adults, adults living in non-metropolitan areas, and adults with lower educational attainment, with lower income and without insurance; all groups with higher risk for COVID-19 morbidity and mortality (24). This is like the rate of vaccine hesitancy in children according to a study by Alfieri et al. in Sept. 2021. This study looked at parents with children <18 years. old in Chicago and Cook County Illinois and found that approximately 33% of parents reported vaccine hesitancy with their child. Like in the Overhage commentary, the groups with the higher rate of vaccine hesitancy included non-Hispanic black parents, publicly insured parents, and parents with a lower income (25).

There is limited data on the rates of the COVID-19 vaccination in CYSHCN, however, there are studies that look at the overall vaccine hesitancy with CYSHCN. A study done by Bonsu et al. looked at vaccine hesitancy among parents whose children have autism spectrum disorder (ASD) compared to parents whose children did not have ASD. They found that 23.6% of parents in this study whose children had ASD were vaccine hesitant. This is compared to a referenced study where they looked general vaccine hesitancy among parents in the same medical institution and found that 8.2% were vaccine hesitant. Most of the vaccine hesitancy in the parents of children with ASD revolved around parents’ belief about the causes of these children's developmental delays including “will of God” and “toxins present in the vaccines” (26). Therefore, there is hesitancy in this group regarding vaccines, which only prolongs the difficulty these families have faced during the pandemic.

## Conclusion

Thus far, the COVID-19 pandemic had a devastating impact on CYSHCN and their families. Not only were CYSHCN more likely to get a severe case of COVID-19, but due to their complexity, they were not able to easily access medical care. Their developmental progress was either delayed due to physical, occupational, or speech therapists canceling services secondary to concerns of spread or placed as an increased burden on the caregivers to aid them during virtual sessions. Those who received only school based services may have had no services at all. A number of parents remained vaccine hesitant even for their vulnerable child. Finally, stay at home orders and the closing of schools and daycare centers placed an increased mental health burden on the children as their daily routines were disrupted. Not having access to their child's usual supports also resulted in their caregivers having increased child care and educational responsibilities with lack of parents’ own supports. There was some light at the end of the tunnel however, particularly among parents who were able to cope well themselves and also assist their children in coping throughout the pandemic and the reopening of the services that these children need. In the United States, many health care centers were able to rapidly pivot to telehealth services and generate new types of home based care supports. These services are also under investigation for improvement in access, decreased burden of transportation, and health care disparities. Studies of the above mentioned data set with specific emphasis on CYSHCN are needed.
